# The Assessment of Serum Fibronectin Levels as a Potential Biomarker for the Severity of Drug-Sensitive Pulmonary Tuberculosis: A Pilot Study

**DOI:** 10.3390/diagnostics14010050

**Published:** 2023-12-25

**Authors:** Shreedhar Kulkarni, Sumalatha Arunachala, Sindaghatta Krishnarao Chaya, Rekha Vaddarahalli ShankaraSetty, Medha Karnik, Nidhi Bansal, Sukanya Ravindran, Komarla Sundararaja Lokesh, Mikash Mohan, Mohammed Kaleem Ullah, Jayaraj Biligere Siddaiah, Padukudru Anand Mahesh

**Affiliations:** 1 Department of Respiratory Medicine, JSS Medical College, JSS Academy of Higher Education and Research, Mysore 570015, Indiaa.suma86@gmail.com (S.A.); rekha.setty23@gmail.com (R.V.S.); sukanya.hema29may@gmail.com (S.R.);; 2 Public Health Research Institute of India, Mysore 570020, India; 3 Department of Critical Care Medicine, Adichunchanagiri Institute of Medical Sciences, Bellur 571448, India; 4 Centre for Excellence in Molecular Biology and Regenerative Medicine (A DST-FIST Supported Center), Department of Biochemistry (A DST-FIST Supported Department), JSS Medical College, JSS Academy of Higher Education and Research, Mysore 570015, India; medhakarnik07@gmail.com (M.K.);; 5 Division of Infectious Disease and Vaccinology, School of Public Health, University of California, Berkeley, CA 94720, USA

**Keywords:** fibronectin, sputum positive PTB, *Mycobacterium tuberculosis*, mycobacterial load, disease severity

## Abstract

Background: Tuberculosis (TB) is a global health burden caused by *Mycobacterium tuberculosis* (Mtb) infection. Fibronectin (Fn) facilitates Mtb attachment to host cells. We studied the Fn levels in smear-positive TB patients to assess its correlation with disease severity based on sputum smears and chest X-rays. Methods: Newly detected consecutive sputum AFB-positive pulmonary TB patients (*n* = 78) and healthy control subjects (*n* = 11) were included. The mycobacterial load in the sputum smear was assessed by IUATLD classification, ranging from 0 to 3. The severity of pulmonary involvement was assessed radiologically in terms of both the number of zones involved (0–6) and as localized (up to 2 zones), moderate (3–4 zones), or extensive (5–6 zones). The serum human fibronectin levels were measured by using a commercially available enzyme-linked immunosorbent assay (ELISA) kit (Catalogue No: CK-bio-11486, Shanghai Coon Koon Biotech Co., Ltd., Shanghai, China). Results: The PTB patients showed lower Fn levels (102.4 ± 26.7) compared with the controls (108.8 ± 6.8), but they were not statistically significant. Higher AFB smear grades had lower Fn levels. The chest X-ray zones involved were inversely correlated with Fn levels. The Fn levels, adjusted for age and gender, decreased with increased mycobacterial load and the number of chest radiograph zones affected. A Fn level <109.39 g/mL predicted greater TB severity (sensitivity of 67.57% and specificity of 90.38%), while a level <99.32 pg/mL predicted severity based on the chest radiology (sensitivity of 84.21% and specificity of 100%). Conclusions: The Fn levels are lower in tuberculosis patients and are negatively correlated with severity based on sputum mycobacterial load and chest radiographs. The Fn levels may serve as a potential biomarker for assessing TB severity, which could have implications for early diagnosis and treatment monitoring.

## 1. Introduction

Tuberculosis (TB) is a communicable disease that poses a significant global health burden, contributing to a high incidence of illness and mortality worldwide [1]. Prior to the COVID-19 pandemic, TB ranked as the leading cause of death from a single infectious agent, surpassing HIV and AIDS. Disturbingly, recent trends indicate a reversal of the declining trajectory observed between 2005 and 2019, with an increase in TB-related deaths reported between 2019 and 2021 [1]. In 2021, an estimated 1.4 million deaths occurred among HIV-negative individuals, while 187,000 deaths were recorded among those with HIV, resulting in a combined total of 1.6 million deaths [1]. Additionally, the TB incidence rate, measured as new cases per 100,000 people per year, experienced a 3.6% increase in 2021, reversing the previously observed decline of approximately 2% per year over the past two decades [1]. In India, the increase in the TB incidence rate is primarily attributed to several key factors, including patient non-adherence to treatment that can result in the development of drug-resistant TB strains, further weakening the patient’s immune system [2]. India bears a substantial burden of TB cases globally, with the highest number of incident cases reported annually. TB-related mortality ranks as the third leading cause of years of life lost (YLLs) in the country [3]. Thus, the burden of TB remains a significant public health concern both globally and specifically in India, and timely diagnosis and treatment of TB are vital for reducing the associated morbidity and mortality rates.

The initial phase of primary infection in pulmonary tuberculosis involves the attachment of *Mycobacterium tuberculosis* (Mtb) organisms to alveolar macrophages (AMs) [4,5,6,7,8,9,10]. Mtb interacts with various receptors on the surface of host cells and extracellular matrix (ECM) components, such as fibronectin, collagen, and vitronectin, which serve as binding sites for the adhesins of the tubercle bacilli [10]. Among these components, fibronectin (Fn) plays a critical role, as it facilitates the adherence of a larger number of Mtb bacilli and their subsequent entry into host cells [6]. Fibronectin (Fn) serves as a constituent of the extracellular matrix (ECM) and plays diverse roles encompassing cell adhesion, proliferation, mobility, and specialization [11]. Fn is composed of multiple repetitions of three analogous modules (FnI, FnII, and FnIII), which assemble into various functional domains: an initial N-terminal domain (NTD), a gelatin-binding domain (GBD), a cell-binding domain (CBD), a 40 kDa domain housing heparin-binding domain II (Hep-2) and FnIII-15, and fibrin-binding domain II [11]. Bacterial adhesins frequently target Fn, leading to the identification of numerous bacterial adhesins that bind to Fn [11]. Mycobacteria possess a range of proteins that bind to fibronectin (Fn), with the most pivotal one being the antigen 85 complex. This complex comprises three proteins—85A, 85B, and 85C—coded by distinct genes situated at various locations within the mycobacterial genome [11,12]. Animal studies have demonstrated the significance of Fn-binding protein expression in bacterial virulence, highlighting that decreased adhesion correlates with a reduction in virulence [12]. Notably, the presence of exogenous Fn (at a concentration of 10 μg/mL) significantly enhances the attachment of Mtb to AMs [7]. Consequently, fibronectin emerges as an essential integrin in the pathogenesis of tuberculosis (TB).

To date, investigations regarding the role of fibronectin in the pathogenesis of tuberculosis (TB) have primarily relied on in vitro studies or animal models [5,7,10,13]. Adhesin-based antibodies targeting Fn have been tried in animal models to prevent bacterial colonization [12]. Also, it has been proposed that Fn-binding proteins have the potential to serve as vaccine components [12]. However clinical trials specifically examining fibronectin levels in patients with pulmonary TB are scarce [14,15,16]. If its clinical significance is established, fibronectin could be explored as a potential target molecule for drug therapy aimed at preventing TB transmission. Therefore, in this pilot study, we aim to assess the fibronectin levels in individuals with smear-positive pulmonary tuberculosis and determine whether any association exists between the fibronectin levels and the severity of tuberculosis (based on chest X-rays and sputum smears) in patients admitted to a tertiary care center in India.

## 2. Materials and Methods

This pilot study was conducted at the Department of Respiratory Medicine of JSS Medical College & Hospital, a university-affiliated 1800 bed tertiary care hospital in Mysuru, India, to evaluate the potential of serum fibronectin levels as a biomarker for assessing the severity of tuberculosis (TB). The study was approved by the Institutional Ethics Committee of JSS Medical College in Mysuru (Approval Number: JSS/MC/PG/5156/2020-21; dated 22 January 2021). All procedures of the human study (cohort) followed Indian Council of Medical Research (ICMR) guidelines. Written informed consent was obtained from either the patient or his or her legal guardian.

A diagnosis of pulmonary TB was established by the pulmonologist and confirmed with a positive sputum smear as per the National Tuberculosis Elimination Program (NTEP) guidelines [17]. An experienced pulmonologist and radiologist reviewed the chest X-rays to grade the severity. Furthermore, the following information was collected: age, sex, weight, BMI, medical history including comorbidities, and chest X-ray findings.

### 2.1. Definitions

In this study, the severity of the diseases was based on the mycobacterial load in sputum through an acid-fast bacilli smear and the number of zones, and the type of lesion was based on chest radiographs. Quantiferon testing and mycobacterial cultures, which are not routinely performed under the national control program, were not conducted.

No lesion was defined as a chest radiograph with no abnormal findings or a lesion or cavity in any lung fields. A cavity was defined as a chest radiograph with hollow or fluid-filled spaces in the lung tissue, indicating pathologies like abscesses or infections [18]. Consolidation was defined as radiographs with solid lung areas due to fluid or substance filling, associated with conditions such as pneumonia or tumors [18].

The initial assessment of the disease’s extent in the chest X-ray (CXR) was determined by evaluating the number of lung zones affected. Each lung was divided into three zones, including the upper, middle, and lower regions. Disease involvement in one or two of these zones was categorized as localized disease, while three or four zones indicated a state of moderate disease. Extensive disease was defined by the presence of pathology in five or all six lung zones. The smoking habit was classified as smokers or non-smokers. Non-smokers were defined as subjects who had never smoked. Smokers were defined as subjects who continued to smoke [19]. An experienced pulmonologist, radiologist, and microbiologist was defined as a certified specialist with at least 3 years of formal training in the relevant field and more than 10 years of clinical experience. Diabetes was defined as a fasting plasma glucose level ≥126 mg/dL (7.0 mmol/L). Fasting was defined as no caloric intake for at least 8 h. In a patient with classic symptoms of hyperglycemia, a random plasma glucose level ≥200 mg/dL (11.1 mmol/L) was defined as diabetes [20].

### 2.2. Inclusion and Exclusion Criteria

- Inclusion criteria for cases:

Newly detected sputum-positive pulmonary tuberculosis patients were included. Only drug-sensitive cases of PTB were included.

- Exclusion criteria for cases:

Patients with previous TB disease and patients with comorbidities known to affect fibronectin levels like malignancy, receiving anticoagulation, a history with a recent surgery (within the last month), burns, hepatic failure, inflammatory diseases (connective tissue and autoimmune disorders), disseminated intravascular coagulation (DIC), sepsis, dermatological disorders (eczema, psoriatic arthritis, and dermatitis herpetiformis), thrombotic thrombocytopenic purpura, and pregnancy, were excluded from the study. Drug-resistant PTB and HIV-positive subjects were excluded from the study.

- Inclusion criteria for the controls:

Healthy adults aged more than 18 years who gave informed consent were included in the study.

- Exclusion criteria for the controls:

Patients who were pregnant or had a history of recent burns, a history with a recent surgery, a family history of connective tissue disorders or autoimmune disease, or recent trauma were excluded from the study.

### 2.3. Chest X-ray Assessment

Chest X-rays were taken from the posterior-anterior view with adequate exposure to assess the number of zones involved and the type of lesion. The chest X-ray was divided into six zones by drawing two lines: one at the inferior wall of the arch of the aorta and another at the inferior wall of the right inferior pulmonary vein. The chest X-rays were assessed by experienced pulmonologists for the number and type of lesions in various fields.

### 2.4. Sputum Examination

The Ziehl–Neelsen staining method was employed to detect *Mycobacterium tuberculosis* in the sputum samples. Microscope slides were labeled and inoculated with sputum specimens, followed by heat fixation to ensure adherence. Carbol fuchsin, the primary stain, was applied to cover the smears, and controlled heating was performed to facilitate stain penetration. Subsequent decolorization using acid-alcohol and immediate rinsing were executed to remove excess staining. Counterstaining with methylene blue ensued, followed by air drying of the slides. Microscopic examination under oil immersion revealed the presence of acid-fast bacilli characterized by bright red staining against a contrasting blue background, indicative of the potential presence of *Mycobacterium tuberculosis*, and it was classified according to the International Union Against Tuberculosis and Lung Disease (IUATLD) classification [21]. The slides were read by an experienced microbiologist and were reported according to IUATLD classification.

“No AFB” signifies that no AFB was detected in 100 fields of view; “1–9 AFB” means there were 1–9 AFB observed in 100 oil immersion fields; “1+” indicates 10–99 AFB in 100 fields; “2+” denotes 1–10 AFB per field in 50 fields of view; and “3+” represents a high concentration with more than 10 AFB per field in 20 fields.

### 2.5. Cartridge-Based Nucleic Acid Amplification Test (CB-NAAT, GeneXpert)

As per the National Tuberculosis Elimination Program guidelines, the sputum was also subjected to a CB-NAAT analysis using Cepheid from GeneXpert^®^ System. The specimen was combined with a double volume of Xpert *Mycobacterium tuberculosis*/rifampicin (MTB/Rif) sample reagent and agitated for 10 s, followed by a 5 min incubation period at room temperature. Subsequently, 2 mL of the prepared sample was transferred into the Xpert MTB/Rif cartridge and inserted into the machine. Within the cartridge, the sample merged with the sample processing control (SPC), and a filter within the system captured both the sample and the SPC. Through ultrasonic lysis, bacterial cell DNA, if present, was released. The eluted DNA interacted with the dried bead reagents contained in the cartridge. Real-time PCR and detection processes took place within the system, culminating in results available for viewing and printing within an average duration of 1 h and 52 min. Only patients with “Rifampicin resistance not detected” were enrolled in the study.

### 2.6. Collection of Blood for Analysis

Five milliliters of peripheral blood was collected from the patients by venipuncture and centrifuged at 3000 rpm for 10 min within 2 h after the collection of blood to isolate serum, which was stored at −80 °C for further analysis, and data from a hematological panel, conducted using a Sysmex XN 1000 automated blood analyzer (Sysmex Corp., Kobe, Japan), were recorded.

### 2.7. Enzyme-Linked Immunosorbent Assay

The serum human fibronectin levels were measured by using a commercially available enzyme-linked immunosorbent assay (ELISA) kit (human fibronectin (FN), Catalogue No: CK-bio-11486, Shanghai Coon Koon Biotech Co., Ltd., Shanghai, China) according to the manufacturer’s instructions. The kit utilized a double antibody sandwich technique and had a detection range from 50 ng/mL to 1600 ng/mL, with a sensitivity of 10 ng/mL. The absorbance was read on an iMark^TM^ Microplate Absorbance Reader (Bio-Rad, Inc., Hercules, CA, USA) at a wavelength of 450 nm.

### 2.8. Statistical Analysis

The statistical analysis was performed using jamovi (v2.2.5., The jamovi Project, SYD, AUS). Descriptive and inferential statistical methods were employed to compare between the normal and TB patients. The normality of the data was assessed using the Shapiro–Wilk test. Continuous variables were presented as the mean ± SD (min-max), and the categorical variables were presented as numbers (%). Analysis of variance (ANOVA) was used to compare the mean values among different groups, while the Student’s *t* test and chi-squared test were utilized to determine the significance of study parameters between two groups. A *p* value of less than 0.05 was considered statistically significant. Linear regression was employed to assess the association between the fibronectin levels and TB severity based on the sputum smear and chest X-ray findings. Receiver operating characteristic (ROC) analysis was performed using the calculated values (determined by Youden’s index) for the area under the curve (AUC), sensitivity, specificity, and odds ratio, and the optimal cut-off values of fibronectin for differentiating between an AFB less than two and more than two, as well as the number of zones involved in the chest X-ray (less than three and more than three), were calculated.

## 3. Results

The demographic parameters examined included age, sex, weight, smoking status, fibronectin levels, total protein levels, albumin levels, red blood cell count, and hemoglobin levels (Table 1). The results showed that there was no significant difference in age between the control subjects and tuberculosis patients (*p* = 0.1681). In terms of sex distribution, both groups had a similar proportion of males and females (*p* = 0.5342). However, a significant difference was observed in weight, with the control subjects having a higher mean weight (72.5 kg ± 0.8) compared with the tuberculosis patients (55.6 kg ± 10.9). Smoking status did lead the groups to differ significantly (*p* = 0.7082), with approximately 58.4% of the participants overall being non-smokers. The fibronectin levels were lower in the PTB patients (102.4 ± 26.7) when compared with the normal group (108.8 ± 6.8). However, it did not achieve statistical significance. Analysis of the fibronectin levels was conducted within subgroups (overall study population, controls, and cases), considering various factors such as age (Table S1), smoking status (Table S2), diabetes (Table S3), AFB score (Table S4), and the number of chest X-ray zones (Table S5) contributing to disease severity.

While the difference in total protein levels approached significance (*p* = 0.0941), there was a significant difference in albumin levels between the control subjects (3.9 g/dL ± 0.5) and the tuberculosis patients (3.3 g/dL ± 0.5) (*p* < 0.0011). The mean red blood cell count exhibited a trend toward significance (*p* = 0.0591), with the control subjects having a lower count (97.3 cells/μL ± 34.1) compared with the tuberculosis patients (164.0 cells/μL ± 114.4). Finally, there was a significant difference in the mean hemoglobin levels between the control subjects (14.7 g/dL ± 2.2) and the tuberculosis patients (11.5 g/dL ± 1.9) (*p* < 0.001).

Regarding the AFB smear results, the mean fibronectin level for patients with 1+ AFB was 118.5 ng/mL (±26.9), which was significantly higher compared with the patients with 2+ AFB (97.7 ng/mL ± 14.9) and 3+ or 4+ AFB (91.1 ng/mL ± 28.7) (*p* < 0.001) (Table 2). In terms of the number of zones involved in the chest X-rays, the mean fibronectin level increased as the number of zones involved increased. The subgroup with the number of zones involved had a mean fibronectin level of 107.2 ng/mL (±32.2). However, there was a gradual decrease in the mean fibronectin level from 109.8 ng/mL (±29.2) for one zone involved to 58.3 ng/mL (±2.1) for five zones involved (Table 2). The difference in mean fibronectin levels between the subgroups was statistically significant (*p* = 0.0261). When considering random blood sugar levels, the patients with blood sugar levels below 140 mg/dL had a higher mean fibronectin level of 107.3 ng/mL (±22.8) compared with those with blood sugar levels above 140 mg/dL (94.3 ng/mL ± 28.1) (*p* = 0.0231). Regarding smoking status, although the mean fibronectin level was higher in smokers (108.6 ng/mL ± 24.2) compared with non-smokers (99.4 ng/mL ± 25.4), the difference was not statistically significant (*p* = 0.0901).

We performed a subgroup analysis of the fibronectin levels in TB patients with diabetes, and no statistically significant difference was found in the fibronectin levels between the diabetic and non-diabetic groups (Table 3) (*p* = 0.325). No significant difference in fibronectin levels was noted in the TB patients with smoking and diabetes versus the non-smoking TB patients without diabetes (*p* = 0.564). There was no statistically significant association noted in the fibronectin levels among diabetic TB patients versus non-diabetic patients when adjusted for mycobacterial load on sputum AFB smears (*p* = 0.822).

**Figure 1:**
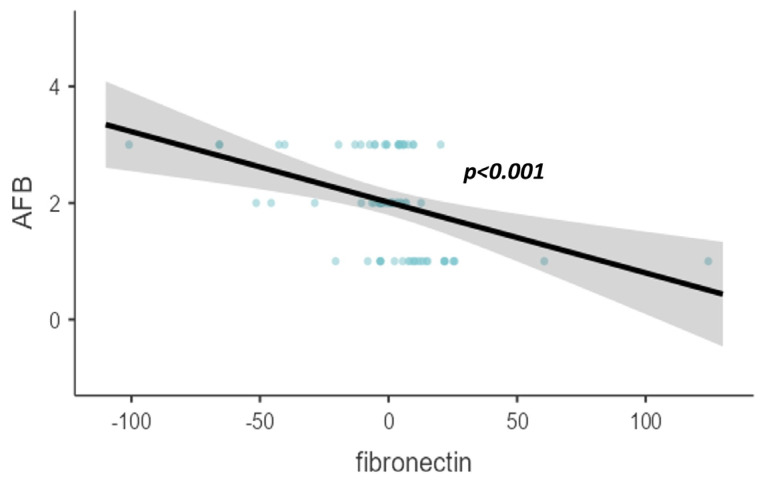
Scatter plot of age- and gender-adjusted fibronectin (pg/dL) levels with AFB.

**Figure 2:**
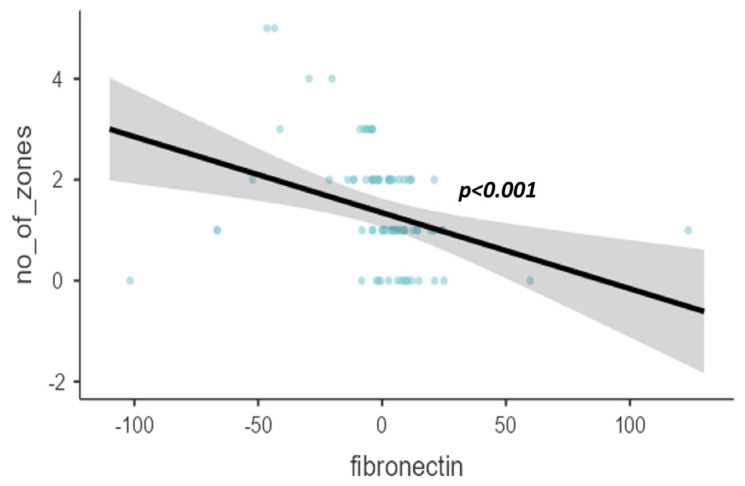
Scatter plot of age- and gender-adjusted fibronectin (pg/dL) levels with the involvement of a number of zones on chest radiographs.

When adjusted to age and gender, fibronectin showed a reducing trend on the scatter plot as the mycobacterial bacterial load in sputum smear examination increased with a significant value (*p* < 0.001), as shown in Figure 1. Similarly, the age- and gender-adjusted fibronectin levels reduced as the number of zones affected by the chest X-ray increased (*p* = 0.001), as shown in Figure 2.

**Figure 3:**
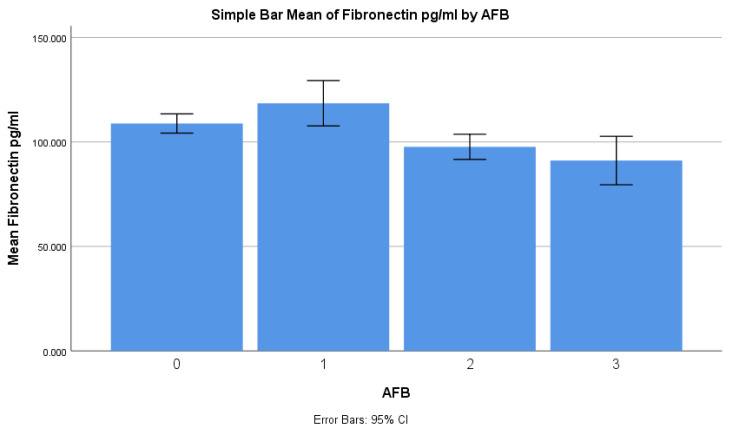
Bar graph depicting mean fibronectin levels against sputum AFB (*p* < 0.001).

**Figure 4:**
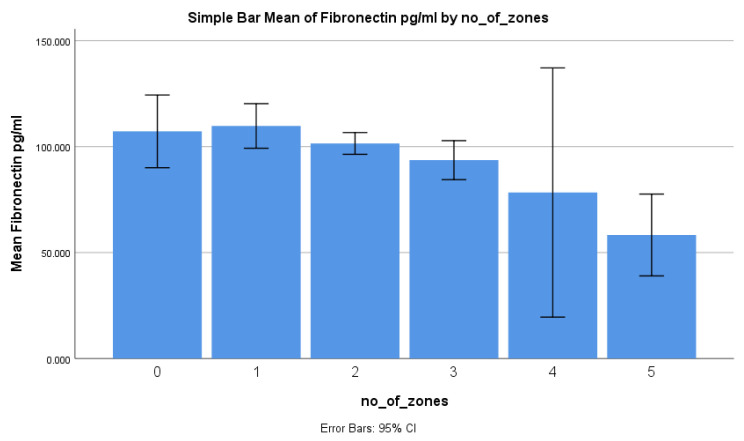
Bar graph depicting mean fibronectin levels against the involvement of a number of zones in a chest X-ray (*p* = 0.0261).

A comparison of the serum fibronectin levels with different bacterial loads based on sputum AFB showed a fall in the levels of fibronectin as the bacterial load increased (Figure 3), and the serum fibronectin levels reduced as the number of zones involved in the chest X-ray increased (Figure 4).

**Figure 5:**
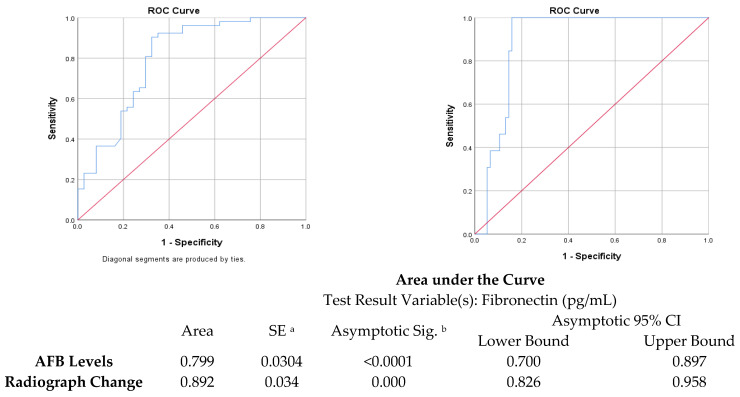
ROC curves for predicting TB severity based on fibronectin levels. ^a^ “Nonparametric” assumption was performed. ^b^ Null hypothesis: true area = 0.5. SE = standard error; CI = confidence interval.

The ROC curve was obtained after dividing the study group into subjects with AFB levels less than two and more than two. The curve showed an area under the curve of 0.799 with an error of 0.0304, which was statically significant (*p* < 0.0001), as well as a sensitivity and specificity of 67.57% and 90.38%, respectively (Figure 5). Youden’s index was 0.58, suggesting a moderate balance between the sensitivity and specificity. Similarly, the subjects were divided into subjects with less than three zones involved and three or more zones involved, which showed an area under the curve of 0.892, a standard error of 0.034, a significance of *p* = 0.0001, and a sensitivity and specificity of 84.21% and 100%, respectively (Table 4). Youden’s index was 0.842, suggesting a good balance between the sensitivity and specificity.

In our study, we conducted a multiple linear regression analysis to explore the relationships between various predictor variables and the severity of tuberculosis (TB), which we assessed using two different measures: mycobacterial load in the sputum and chest X-ray (CXR) severity. Our analysis revealed that fibronectin demonstrates a statistically significant and independent association with TB severity, as assessed by the mycobacterial load in the sputum (*p* < 0.001) and CXR severity (*p* = 0.003). This finding underscores the role of fibronectin as a significant predictor of TB severity using both of these assessment methods. However, several other variables, including age, gender, albumin, hemoglobin (HB), and the neutrophil-to-lymphocyte ratio (NLR), do not appear to have a statistically significant association with TB severity. These variables, while considered in our analysis, did not exhibit a significant impact on the severity of TB as assessed by the mycobacterial load or CXR. Interestingly, we observed that patients with lower body weights exhibited increased TB severity, as evidenced by greater involvement of lung zones on the chest X-rays (*p* = 0.044). This suggests a potential relationship between a lower body weight and more severe TB manifestations in CXR.

## 4. Discussion

The present study evaluated whether the fibronectin levels in sputum-positive pulmonary TB patients are lower when compared with normal individuals. It also evaluated the relationship between fibronectin and the severity of TB as judged by the sputum smear mycobacterial load and the number of zones involved in chest X-rays before initiation of treatment. We observed that the fibronectin levels were lower in patients with pulmonary TB compared with normal individuals, but the difference was not statistically significant. Similarly, it was higher in smokers compared with non-smokers with TB, but again, the differences were not statistically significant. There was a negative correlation between TB severity (based on the sputum smear load and number of zones involved in the chest X-ray) and the fibronectin levels, which was statistically significant (*p* < 0.001). The ROC curve was obtained after dividing the study group into subjects with AFB levels less than two and more than two. A fibronectin level of less than 109.39 pg/mL was able to predict greater severity of pulmonary tuberculosis, with a sensitivity of 67.57% and specificity of 90.38%. A fibronectin level of 99.32 pg/mL was able to predict greater severity of pulmonary tuberculosis based on chest radiology, with a sensitivity of 84.21% and specificity of 100%. These findings underscore the complexity of fibronectin dynamics in TB infection and its potential role in the host immune response. We observed a significant and independent association of fibronectin with TB severity when assessed by mycobacterial load and CXR severity. Additionally, a lower body weight was found to be associated with increased TB severity in CXR.

An experimental study conducted to investigate the role of fibronectin in the attachment of *Mycobacterium tuberculosis* to alveolar macrophages revealed important insights into the dynamics of this process [6,7]. The findings demonstrated that in the absence of fibronectin, the attachment of *M. tuberculosis* to alveolar macrophages decreased, indicating that fibronectin plays a crucial role in modulating the attachment process. Conversely, when fibronectin was present, attachment was found to increase in a concentration-dependent manner, highlighting the significance of the fibronectin concentration in influencing the attachment of *M. tuberculosis*. This association suggests that as the number of zones involved in tuberculosis lesions increases, the mycobacterial load increases, and subsequent fibronectin utilization by alveolar macrophages may lead to decreased fibronectin levels. We observed that the fibronectin levels were significantly reduced as the number of bacilli on the sputum microscopy increased and with the increased number of zones involved radiologically.

There is only one clinical study on serum fibronectin levels in patients with TB in the last two decades. A study conducted in China in 2014, which is similar to our own, explored the association between fibronectin levels and tuberculosis (TB) patients compared to individuals without pulmonary tuberculosis (TB). The results indicated that the patients with TB had significantly lower levels of fibronectin compared with those without pulmonary TB, similar to the findings of our study [16]. Furthermore, the study revealed that the fibronectin levels were lower in cases of clinically severe pulmonary tuberculosis compared with minimal and moderate pulmonary tuberculosis. However, no significant difference in fibronectin levels was observed when comparing the increasing IUATLD grading scale of acid-fast bacilli (AFB), a common diagnostic marker for mycobacterial load, among the study participants [16]. The relationship between the fibronectin levels and different types of TB lesions, such as cavity or consolidation, as well as single lung lesions or double lung lesions was also studied. Interestingly, no significant difference in fibronectin levels was found between these lesion types [16]. We did not find any differences in fibronectin levels between different types of radiological lesions.

Given that malnutrition enhances TB susceptibility, understanding how weight and tuberculosis (TB) interact is essential. Studies have shown that people are more likely to develop tuberculosis if they have a low BMI or less muscle and fat mass [22,23,24,25,26]. Weight loss is a common side effect of TB, and a sizable portion of TB patients experience it [27,28]. The risk of premature mortality during TB therapy is also markedly increased by severe malnutrition [28,29]. Similarly, our study found increased severity of TB in CXR in patients with lower body weights. It is important to address malnutrition in order to prevent and manage TB effectively, underscoring the complex relationship between TB and weight.

We observed significantly lower hemoglobin levels among the TB patients compared with the controls. There are several mechanisms related to lower hemoglobin levels in patients with tuberculosis. Low hemoglobin (Hb) levels in tuberculosis (TB) are mainly due to poor nutrition and the effect of TB itself [30]. In addition, TB-related anemia stems from malabsorption and chronic inflammation, triggered by systemic inflammation and lung damage [31]. Anemia of chronic disease, which is observed in TB, results from inflammation-related factors affecting the erythrocyte lifespan, iron incorporation, and erythropoietin sensitivity [32,33]. *Mycobacterium tuberculosis* (MTB)-associated tissue inflammation results in the release of cytokines (IL-1, TNF-α, and IL-10), lipid mediators, and IFN-γ [34]. TB-induced inflammation disrupts iron homeostasis, causing iron retention in the reticuloendothelial system, limiting availability for erythroid cells and leading to iron-restricted erythropoiesis [35]. IFN-γ, LPS, and TNF-α enhance DMT1 expression, increasing iron absorption by macrophages, while suppressing ferroportin prevents iron release [36,37]. Hepcidin, induced by LPS and IL-6, contributes to iron dysregulation, hypoferremia, and inflammatory anemia [38]. Inflammation disrupts the usual regulation of hepcidin by iron levels; instead, IL-6 activation enhances hepcidin synthesis [39]. Hepcidin hinders iron release from macrophages, the liver, and the duodenum by binding and downregulating ferroportin receptors. This process leads to iron sequestration in the reticuloendothelial system [40,41]. Iron retention in the reticuloendothelial system is considered a host defense strategy against *Mycobacterium tuberculosis* (MTB), since iron is vital for its growth [42]. The inflammatory cytokines TNF-α, IL-1, and endotoxin reduce the RBC lifespan and plasma iron incorporation. Iron metabolism in inflammatory diseases restricts tissue iron release, lowers the serum iron TIBC, and elevates serum ferritin. A transferrin decline results from increased protein catabolism. This altered iron metabolism is considered part of the host defense system against invading pathogens, potentially mediated by inflammatory cytokines [43]. The decline in transferrin concentration is primarily attributed to heightened protein catabolism [44].

Several studies have shown that fibronectin levels are increased in patients with diabetes, especially in those with uncontrolled sugars [45,46,47,48,49]. Our study is the first to evaluate the association of fibronectin levels in diabetic TB patients. We could not find any difference in fibronectin levels in diabetic TB patients compared with non-diabetic patients with TB. This could be due to the opposing effect of diabetes and TB on the fibronectin levels, a low sample size, or a lack of data on HBA1C (and hence a lack of data on long-term diabetes control in patients with both TB and diabetes). Further studies are required to explore the combined effect of diabetes and TB on fibronectin levels.

To the best of our knowledge, this is the first study to examine fibronectin levels in sputum-positive pulmonary TB patients in India. The strengths of our study include well-defined inclusion and exclusion criteria and a clear methodology including correlation with severity of tuberculosis based on the mycobacterial load and radiologic severity of tuberculosis. Limitations of our study include the small sample size, small number of controls, lack of serial fibronectin levels to evaluate changes over time, and a lack of evaluation of severity based on CT and testing for latent TB and non-tuberculous pneumonia as an additional comparator group.

## 5. Conclusions

The fibronectin levels were similar in tuberculosis patients compared to the healthy controls. However, they were negatively correlated with the severity of pulmonary tuberculosis based on sputum mycobacterial load and chest radiograph severity and were significantly lower in severe disease compared with mild disease. The Fn levels may serve as a potential biomarker for assessing TB severity, which could have implications for early diagnosis and treatment monitoring. Further clinical studies on a larger scale are needed to confirm our findings. It will be useful to include controls without latent TB infections, and controls with latent TB infections and non-tuberculosis pneumonia to confirm the validity of our findings that fibronectin can serve as a useful biomarker for severe tuberculosis.

## Figures and Tables

**Table 1:** Comparison of demographic and clinical characteristics between control subjects (*n* = 11) and patients with tuberculosis (*n* = 78).

	Normal (*n* = 11)	PTB (*n* = 78)	Total (*n* = 89)	*p* Value
Age (years)				0.1681 ^†^
Mean (SD)	40.3 ± 15.3	47.0 ± 15.1	46.2 ± 15.2	
Range	26.0–75.0	19.0–75.0	19.0–75.0	
Gender				0.5342 ^†^
Female	3.0 (27.3%)	15.0 (19.2%)	18.0 (20.2%)	
Male	8.0 (72.7%)	63.0 (80.8%)	71.0 (79.8%)	
Weight (kg)				<0.001 *
Mean (SD)	72.5 ± 0.8	55.6 ± 10.9	57.7 ± 11.9	
Range	58.0–84.0	36.0–84.0	36.0–84.0	
Smoking				0.708 ^†^
No	7.0 (63.6%)	45.0 (57.7%)	52.0 (58.4%)	
Yes	4.0 (36.4%)	33.0 (42.3%)	37.0 (41.6%)	
Fibronectin (ng/mL)				0.431 *
Mean (SD)	108.8 ± 6.8	102.4 ± 26.7	103.2 ± 25.2	
Range	94.9–118.0	1.5–226.8	1.5–226.8	
Total Protein (g/dL)				0.094 *
Mean (SD)	7.0 ± 0.4	6.6 ± 0.7	6.6 ± 0.7	
Range	6.2–7.6	5.2–8.5	5.2–8.5	
Albumin (g/dL)				<0.001 *
Mean (SD)	3.9 ± 0.5	3.3 ± 0.5	3.4 ± 0.5	
Range	3.4–4.9	2.1–4.6	2.1–4.9	
RBS (mg/dL)				0.0591 *
Mean (SD)	97.3 ± 34.1	164.0 ± 114.4	155.7 ± 109.9	
Range	45.0–170.0	58.0–643.0	45.0–643.0	
HB (g/dL)				<0.001 *
Mean (SD)	14.7 ± 2.2	11.5 ± 1.9	11.9 ± 2.2	
Range	10.2–18.3	8.0–17.1	8.0–18.3	

* ANOVA. ^†^ Chi-squared test. RBS = random blood sugar; HB: hemoglobin; PTB: pulmonary tuberculosis.

**Table 2:** Fibronectin levels in different subgroups of tuberculosis patients: Associations with acid-fast bacilli smear, chest X-ray, random blood sugar, smoking status, and type of lesion on radiograph.

Fibronectin	Mean (pg/dL) ± SD	*p* Value
Normal	108.8 ± 6.8	0.4311 *
PTB	102.4 ± 26.7	
Based on AFB		<0.001 *
1+	118.5 ± 26.9	
2+	97.7 ± 14.9	
3+ or 4+	91.1 ± 28.7	
Based on the number of zones		0.0261 *
0	107.2 ± 32.2	
1	109.8 ± 29.2	
2	101.5 ± 13.2	
3	93.6 ± 12	
4	78.3 ± 6.5	
5	58.3 ± 2.1	
Smoking		0.0901 *
No	99.4 ± 25.4	
Yes	108.6 ± 24.2	
Based on type of lesion on radiograph		0.725 *
Normal	107.2 ± 32.2	
Cavity	102.1 ± 15.2	
Consolidation	102.4 ± 26.1	

* ANOVA. PTB = pulmonary tuberculosis; AFB = acid-fast bacilli.

**Table 3:** Comparative analysis of fibronectin levels in diabetic versus non-diabetic TB patients.

Study Groups	<200 mg/dL	>200 mg/dL	Total	*p* Value
Total (*n*)	57	21	78	
Male (*n*, %)	42.0 (73.7%)	21.0 (100.0%)	63.0 (80.8%)	0.009 ^1^
Female (*n*, %)	15.0 (26.3%)	0.0 (0.0%)	15.0 (19.2%)	
Fibronectin (pg/mL) (mean ± SD)	104.2 ± 28.1	97.5 ± 22.4	102.4 ± 26.7	0.325 ^2^
Age < 40 (*n*, %)	22.0 (38.6%)	4.0 (19.0%)	26.0 (33.3%)	0.104 ^1^
Age > 40 (*n*, %)	35.0 (61.4%)	17.0 (81.0%)	52.0 (66.7%)	
Non-smokers (*n*, %)	34.0 (59.6%)	11.0 (52.4%)	45.0 (57.7%)	0.564 ^1^
Smokers (*n*, %)	23.0 (40.4%)	10.0 (47.6%)	33.0 (42.3%)	
AFB 0 (*n*, %)	-	-	-	0.822 ^1^
AFB 1 (*n*, %)	19.0 (33.3%)	7.0 (33.3%)	26.0 (33.3%)	
AFB 2 (*n*, %)	20.0 (35.1%)	6.0 (28.6%)	26.0 (33.3%)	
AFB 3 (*n*, %)	18.0 (31.6%)	8.0 (38.1%)	26.0 (33.3%)	

^1^ Pearson’s chi-squared test. ^2^ ANOVA. AFB = acid-fast bacilli.

**Table 4:** Cut-off values of fibronectin for the above ROC curves.

	Cut Point	Sensitivity (%)	Specificity (%)	Youden’s Index	AUC
Fibronectin vs. chest X-ray zones	99.3 ng/mL	84.21%	100%	0.842	0.892
Fibronectin vs. sputum AFB	109.4 ng/mL	67.57%	90.38%	0.58	0.799

AUC = area under the curve; ROC = receiver operating characteristic curve; AFB = acid-fast bacilli.

## Data Availability

All data generated or analyzed during this study are included in this published article and are available from the corresponding author upon reasonable request.

## Supplementary Materials

The following supporting information can be downloaded at https://www.mdpi.com/article/10.3390/diagnostics14010050/s1. Table S1: Demographic and clinical characteristics comparison across age groups in overall study population, controls, and cases. Table S2: Demographic and clinical characteristics comparison between non-smokers and smokers in overall study population, controls, and cases. Table S3: Demographic and clinical characteristics comparison between non-diabetic and diabetic patients in overall study population, controls, and cases. Table S4: Demographic and clinical characteristics comparison between AFB scores in overall study population, controls, and cases. Table S5: Demographic and clinical characteristics comparison between number of chest X-ray zones in overall study population, controls, and cases.
