# Supplementary material for: The Assessment of Serum Fibronectin Levels as a Potential Biomarker for the Severity of Drug-Sensitive Pulmonary Tuberculosis: A Pilot Study
Source: Diagnostics (Basel). 2023 Dec 25;14(1):50. doi: 10.3390/diagnostics14010050 (PMC10804257; doi:10.3390/diagnostics14010050)
Supplement: Supplementary file 1 [file diagnostics-14-00050-s001.zip › diagnostics-2686516-supplementary.pdf]

diagnostics

Article

Assessment of Serum Fibronectin Levels as a Potential Biomarker for Severity of Drug Sensitive-Pulmonary Tuberculosis: A Cross-Sectional Study: Supplementary File

Shreedhar Kulkarni [1], Sumalatha Arunachala [1,2,3], Sindaghatta Krishnarao Chaya [1], Rekha V S [1], Medha Karnik [4], Nidhi Bansal [1], Sukanya Ravindran [1], Komarla Sundararaja Lokesh [1], Mikash Mohan [1], Mohammed Kaleem Ullah [4,5], Jayaraj Biligere Siddaiah [1], Padukudru Anand Mahesh [1*]

1. Department of Respiratory Medicine, JSS Medical College, JSS Academy of Higher Education and Research, Mysuru 570015, India
2. Public Health Research Institute of India, Mysuru 570020, India.
3. Department of Critical Care Medicine, Adichunchanagiri Institute of Medical Sciences, Bellur 571448, India
4. Centre for Excellence in Molecular Biology and Regenerative Medicine (A DST-FIST Supported Center), Department of Biochemistry (A DST-FIST Supported Department), JSS Medical College, JSS Academy of Higher Education and Research, Mysore 570015, India.
5. Division of Infectious Disease and Vaccinology, School of Public Health, University of California, Berkeley, CA 94720, USA.

* Correspondence:
Professor Padukudru Anand Mahesh
Department of Respiratory Medicine, JSS Medical College, JSSAHER,
Mysore, Karnataka, India.
Direct line: +91-9448044003
Email: pamahesh@jssuni.edu.in

Citation: To be added by editorial staff during production.

Academic Editor: Firstname Lastname

Received: date
Revised: date
Accepted: date
Published: date

Copyright: © 2023 by the authors. Submitted for possible open access publication under the terms and conditions of the Creative Commons Attribution (CC BY) license (https://creativecommons.org/licenses/by/4.0/).

Table S1: Demographic and clinical characteristics comparison across age groups in overall study population, controls, and cases.

Overall study population

**Study Groups**	**Age<40**	**Age>40**	**Total**	***p*-value**
Total (n)	33	56	89	
Male	24.0 (72.7%)	47.0 (83.9%)	71 (79.8%)	*0.204*[1]
Female	9.0 (27.3%)	9.0 (16.1%)	18 (20.2%)	
Fibronectin (pg/ml) (mean±SD)	107.6±11.2	100.6±30.4	103.2±25.2	*0.203*[2]
Non-Smokers	23.0 (69.7%)	29.0 (51.8%)	52.0 (58.4%)	*0.098*[1]
Smokers	10.0 (30.3%)	27.0 (48.2%)	37.0 (41.6%)	
Non-Diabetic	29.0 (87.9%)	39.0 (69.6%)	68.0 (76.4%)	*0.050*[1]
Diabetic	4.0 (12.1%)	17.0 (30.4%)	21.0 (23.6%)	
AFB 0	7.0 (21.2%)	4.0 (7.1%)	11.0 (12.4%)	*0.076*[1]
AFB 1+	12.0 (36.4%)	14.0 (25.0%)	26.0 (29.2%)	
AFB 2+	6.0 (18.2%)	20.0 (35.7%)	26.0 (29.2%)	
AFB 3+/4+	8.0 (24.2%)	18.0 (32.1%)	26.0 (29.2%)	
No of Zones				*0.474*[1]
0	8.0 (24.2%)	8.0 (14.3%)	16.0 (18.0%)	
1	10.0 (30.3%)	22.0 (39.3%)	32.0 (36.0%)	
2	9.0 (27.3%)	19.0 (33.9%)	28.0 (31.5%)	
3	5.0 (15.2%)	4.0 (7.1%)	9.0 (10.1%)	
4	1.0 (3.0%)	1.0 (1.8%)	2.0 (2.2%)	
5	0.0 (0.0%)	2.0 (3.6%)	2.0 (2.2%)	

Only controls

**Study Groups**	**Age<40**	**Age>40**	**Total**	***p*-value**
Total (n)	07	04	11	
Male	4 (57.1%)	4 (100%)	8 (72.7%)	*0.125*[1]
Female	3 (42.9%)	0 (0)	3 (27.3%)	
Fibronectin (pg/ml) (mean±SD)	109.6±4.0	107.5±11.0	108.8±6.8	*0.646*[2]
Non-Smokers	5.0 (71.4%)	2.0 (50.0%)	7.0 (63.6%)	*0.477*[1]
Smokers	2.0 (28.6%)	2.0 (50.0%)	4.0 (36.4%)	
Non-Diabetic	7.0 (100.0%)	4.0 (100.0%)	11.0 (100.0%)	*0.366*[3]
Diabetic	-	-	-	
AFB 0	7.0 (100.0%)	4.0 (100.0%)	11.0 (100.0%)	*0.366*[3]
No of Zones				*0.366*[3]
0	7.0 (100.0%)	4.0 (100.0%)	11.0 (100.0%)	

Only Patients with TB

**Study Groups**	**Age<40**	**Age>40**	**Total**	***p*-value**
Total (n)	26	52	78	
Male	20.0 (76.9%)	43.0 (82.7%)	63.0 (80.8%)	*0.542*[1]
Female	6.0 (23.1%)	9.0 (17.3%)	15.0 (19.2%)	
Fibronectin (pg/ml) (mean±SD)	107.1±12.4)	100.1±31.3)	102.4±26.7	*0.274*[2]

Non-Smokers	18.0 (69.2%)	27.0 (51.9%)	45.0 (57.7%)	*0.145*[1]
Smokers	8.0 (30.8%)	25.0 (48.1%)	33.0 (42.3%)	
Non-Diabetic	22.0 (84.6%)	35.0 (67.3%)	57.0 (73.1%)	*0.104*[1]
Diabetic	4.0 (15.4%)	17.0 (32.7%)	21.0 (26.9%)	
AFB 0	-	-	-	*0.199*[1]
AFB 1+	12.0 (46.2%)	14.0 (26.9%)	26.0 (33.3%)	
AFB 2+	6.0 (23.1%)	20.0 (38.5%)	26.0 (33.3%)	
AFB 3+/4+	8.0 (30.8%)	18.0 (34.6%)	26.0 (33.3%)	
No of Zones				*0.589*[1]
0	1.0 (3.8%)	4.0 (7.7%)	5.0 (6.4%)	
1	10.0 (38.5%)	22.0 (42.3%)	32.0 (41.0%)	
2	9.0 (34.6%)	19.0 (36.5%)	28.0 (35.9%)	
3	5.0 (19.2%)	4.0 (7.7%)	9.0 (11.5%)	
4	1.0 (3.8%)	1.0 (1.9%)	2.0 (2.6%)	
5	0.0 (0.0%)	2.0 (3.8%)	2.0 (2.6%)	

[1]Pearson's Chi-squared test; [2] ANOVA. AFB: Acid-Fast Bacilli.

Table S2: Demographic and clinical characteristics comparison between non-smokers and smokers in overall study population, controls, and cases.

**Overall study population**				
**Study Groups**	**Non-Smoker**	**Smoker**	**Total**	***p*-value**
Total (n)	52	37	89	
Male	34.0 (65.4%)	37.0 (100.0%)	71.0 (79.8%)	*< 0.001*[1]
Female	18.0 (34.6%)	0.0 (0.0%)	18.0 (20.2%)	
Fibronectin (pg/ml) (mean±SD)	99.4±25.4	108.6±24.2	103.2±25.2	*0.090*[2]
Age<40	23.0 (44.2%)	10.0 (27.0%)	33.0 (37.1%)	*0.098*[1]
Age>40	29.0 (55.8%)	27.0 (73.0%)	56.0 (62.9%)	
Non-Diabetic	41.0 (78.8%)	27.0 (73.0%)	68.0 (76.4%)	*0.520*[1]
Diabetic	11.0 (21.2%)	10.0 (27.0%)	21.0 (23.6%)	
AFB 0	7.0 (13.5%)	4.0 (10.8%)	11.0 (12.4%)	*0.104*[1]
AFB 1+	17.0 (32.7%)	9.0 (24.3%)	26.0 (29.2%)	
AFB 2+	10.0 (19.2%)	16.0 (43.2%)	26.0 (29.2%)	
AFB 3+/4+	18.0 (34.6%)	8.0 (21.6%)	26.0 (29.2%)	
No of Zones				*0.460*[1]
0	10.0 (19.2%)	6.0 (16.2%)	16.0 (18.0%)	
1	19.0 (36.5%)	13.0 (35.1%)	32.0 (36.0%)	
2	13.0 (25.0%)	15.0 (40.5%)	28.0 (31.5%)	
3	7.0 (13.5%)	2.0 (5.4%)	9.0 (10.1%)	
4	1.0 (1.9%)	1.0 (2.7%)	2.0 (2.2%)	
5	2.0 (3.8%)	0.0 (0.0%)	2.0 (2.2%)	
**Only controls**				
**Study Groups**	**Non-Smoker**	**Smoker**	**Total**	***p*-value**

Total (n)	7	4	11	
Male	4.0 (57.1%)	4.0 (100.0%)	8.0 (72.7%)	*0.125*[1]
Female	3.0 (42.9%)	0.0 (0.0%)	3.0 (27.3%)	
Fibronectin (pg/ml) (mean±SD)	106.7±7.1	112.5±5.3	108.8±6.8	*0.193*[2]
Age<40	5.0 (71.4%)	2.0 (50.0%)	7.0 (63.6%)	*0.477*[1]
Age>40	2.0 (28.6%)	2.0 (50.0%)	4.0 (36.4%)	
Non-Diabetic	7.0 (100.0%)	4.0 (100.0%)	11.0 (100.0%)	*0.366*[3]
Diabetic	-	-	-	
AFB 0	7.0 (100.0%)	4.0 (100.0%)	11.0 (100.0%)	*0.366*[3]
No of Zones				*0.366*[3]
0	7.0 (100.0%)	4.0 (100.0%)	11.0 (100.0%)	
**Only Patients with TB**				
**Study Groups**	**Non-Smoker**	**Smoker**	**Total**	***p*-value**
Total (n)	45	33	78	
Male	30 (66.7%)	33 (100%)	63 (80.8%)	*<0.001*[1]
Female	15 (33%)	0 (0)	15 (19.2%)	
Fibronectin (pg/ml) (mean±SD)	98.2±27.0	108.1±25.5	102.4±26.7	*0.108*[2]
Age<40	18.0 (40.0%)	8.0 (24.2%)	26.0 (33.3%)	*0.145*[1]
Age>40	27.0 (60.0%)	25.0 (75.8%)	52.0 (66.7%)	
Non-Diabetic	34.0 (75.6%)	23.0 (69.7%)	57.0 (73.1%)	*0.564*[1]
Diabetic	11.0 (24.4%)	10.0 (30.3%)	21.0 (26.9%)	
AFB 0	-	-	-	
AFB 1+	17.0 (37.8%)	9.0 (27.3%)	26.0 (33.3%)	*0.050*[1]
AFB 2+	10.0 (22.2%)	16.0 (48.5%)	26.0 (33.3%)	
AFB 3+/4+	18.0 (40.0%)	8.0 (24.2%)	26.0 (33.3%)	
No of Zones				*0.479*[1]
0	3.0 (6.7%)	2.0 (6.1%)	5.0 (6.4%)	
1	19.0 (42.2%)	13.0 (39.4%)	32.0 (41.0%)	
2	13.0 (28.9%)	15.0 (45.5%)	28.0 (35.9%)	
3	7.0 (15.6%)	2.0 (6.1%)	9.0 (11.5%)	
4	1.0 (2.2%)	1.0 (3.0%)	2.0 (2.6%)	
5	2.0 (4.4%)	0.0 (0.0%)	2.0 (2.6%)	

[1]Pearson's Chi-squared test; [2] ANOVA. AFB: Acid-Fast Bacilli.

Table S3: Demographic and clinical characteristics comparison between non-diabetic and diabetic in overall study population, controls, and cases

**Overall study population**				
**Study Groups**	**Non-Diabetic**	**Diabetic**	**Total**	***p*-value**
Total (n)	68	21	89	
Male	50.0 (73.5%)	21.0 (100.0%)	71.0 (79.8%)	*0.008*[1]
Female	18.0 (26.5%)	0.0 (0.0%)	18.0 (20.2%)	

Fibronectin (pg/ml) (mean±SD)	105.0±25.8	97.5±22.4	103.2±25.2	*0.235*[2]
Age<40	29.0 (42.6%)	4.0 (19.0%)	33.0 (37.1%)	*0.050*[1]
Age>40	39.0 (57.4%)	17.0 (81.0%)	56.0 (62.9%)	
Non-Smokers	41.0 (60.3%)	11.0 (52.4%)	52.0 (58.4%)	*0.520*[1]
Smokers	27.0 (39.7%)	10.0 (47.6%)	37.0 (41.6%)	
AFB 0	11.0 (16.2%)	0.0 (0.0%)	11.0 (12.4%)	*0.231*[1]
AFB 1+	19.0 (27.9%)	7.0 (33.3%)	26.0 (29.2%)	
AFB 2+	20.0 (29.4%)	6.0 (28.6%)	26.0 (29.2%)	
AFB 3+/4+	18.0 (26.5%)	8.0 (38.1%)	26.0 (29.2%)	
No of Zones				*0.168*[1]
0	15.0 (22.1%)	1.0 (4.8%)	16.0 (18.0%)	
1	25.0 (36.8%)	7.0 (33.3%)	32.0 (36.0%)	
2	17.0 (25.0%)	11.0 (52.4%)	28.0 (31.5%)	
3	7.0 (10.3%)	2.0 (9.5%)	9.0 (10.1%)	
4	2.0 (2.9%)	0.0 (0.0%)	2.0 (2.2%)	
5	2.0 (2.9%)	0.0 (0.0%)	2.0 (2.2%)	
**Only controls**				
**Study Groups**	**Non-Diabetic**	**Diabetic**	**Total**	***p*-value**
Total (n)	11	0	11	
Male	8.0 (72.7%)	-	8.0 (72.7%)	-
Female	3.0 (27.3%)	-	3.0 (27.3%)	-
Fibronectin (pg/ml) (mean±SD)	108.8±6.8	-	108.8±6.8	-
Age<40	7.0 (63.6%)	-	7.0 (63.6%)	-
Age>40	4.0 (36.4%)	-	4.0 (36.4%)	-
Non-Smokers	7.0 (63.6%)	-	7.0 (63.6%)	-
Smokers	4.0 (36.4%)	-	4.0 (36.4%)	-
AFB 0	11.0 (100.0%)	-	11.0 (100.0%)	-
No of Zones	-	-	-	-
0	11.0 (100.0%)	-	11.0 (100.0%)	-
**Only Patients with TB**				
**Study Groups**	**Non-Diabetic**	**Diabetic**	**Total**	***p*-value**
Total (n)	57	21	78	
Male	42.0 (73.7%)	21.0 (100.0%)	63.0 (80.8%)	*0.009*[1]
Female	15.0 (26.3%)	0.0 (0.0%)	15.0 (19.2%)	
Fibronectin (pg/ml) (mean±SD)	104.2±28.1	97.5±22.4	102.4±26.7	*0.325*[2]
Age<40	22.0 (38.6%)	4.0 (19.0%)	26.0 (33.3%)	*0.104*[1]
Age>40	35.0 (61.4%)	17.0 (81.0%)	52.0 (66.7%)	
Non-Smokers	34.0 (59.6%)	11.0 (52.4%)	45.0 (57.7%)	*0.564*[1]
Smokers	23.0 (40.4%)	10.0 (47.6%)	33.0 (42.3%)	
AFB 0	-	-	-	
AFB 1+	19.0 (33.3%)	7.0 (33.3%)	26.0 (33.3%)	*0.822*[1]
AFB 2+	20.0 (35.1%)	6.0 (28.6%)	26.0 (33.3%)	

AFB 3+/4+	18.0 (31.6%)	8.0 (38.1%)	26.0 (33.3%)	
No of Zones				*0.509[1]*
0	4.0 (7.0%)	1.0 (4.8%)	5.0 (6.4%)	
1	25.0 (43.9%)	7.0 (33.3%)	32.0 (41.0%)	
2	17.0 (29.8%)	11.0 (52.4%)	28.0 (35.9%)	
3	7.0 (12.3%)	2.0 (9.5%)	9.0 (11.5%)	
4	2.0 (3.5%)	0.0 (0.0%)	2.0 (2.6%)	
5	2.0 (3.5%)	0.0 (0.0%)	2.0 (2.6%)	

[1]Pearson's Chi-squared test; [2] ANOVA. AFB: Acid-Fast Bacilli.

Table S4: Demographic and clinical characteristics comparison between AFB scores in overall study population, controls, and cases

**Overall study population**					
**Study Groups**	**0**	**1+**	**2+**	**3+/4+**	***p*-value**
Total (n)	11	26	26	26	
Male	8.0 (72.7%)	20.0 (76.9%)	23.0 (88.5%)	20.0 (76.9%)	*0.611[1]*
Female	3.0 (27.3%)	6.0 (23.1%)	3.0 (11.5%)	6.0 (23.1%)	
Fibronectin (pg/ml) (mean±SD)	108.8±6.8	118.5±26.9	97.7±14.9	91.1±28.7	*< 0.001[2]*
Age<40	7.0 (63.6%)	12.0 (46.2%)	6.0 (23.1%)	8.0 (30.8%)	*0.076[1]*
Age>40	4.0 (36.4%)	14.0 (53.8%)	20.0 (76.9%)	18.0 (69.2%)	
Non-Smokers	7.0 (63.6%)	17.0 (65.4%)	10.0 (38.5%)	18.0 (69.2%)	*0.104[1]*
Smokers	4.0 (36.4%)	9.0 (34.6%)	16.0 (61.5%)	8.0 (30.8%)	
Non-Diabetic	11.0 (100.0%)	19.0 (73.1%)	20.0 (76.9%)	18.0 (69.2%)	*0.231[1]*
Diabetic	0.0 (0.0%)	7.0 (26.9%)	6.0 (23.1%)	8.0 (30.8%)	
No of Zones					*< 0.001[1]*
0	11.0 (100.0%)	3.0 (11.5%)	0.0 (0.0%)	2.0 (7.7%)	
1	0.0 (0.0%)	14.0 (53.8%)	9.0 (34.6%)	9.0 (34.6%)	
2	0.0 (0.0%)	6.0 (23.1%)	12.0 (46.2%)	10.0 (38.5%)	
3	0.0 (0.0%)	3.0 (11.5%)	3.0 (11.5%)	3.0 (11.5%)	
4	0.0 (0.0%)	0.0 (0.0%)	1.0 (3.8%)	1.0 (3.8%)	
5	0.0 (0.0%)	0.0 (0.0%)	1.0 (3.8%)	1.0 (3.8%)	
**Only controls**					
**Study Groups**	**0**	**1+**	**2+**	**3+/4+**	***p*-value**
Total (n)	07	-	-	-	-
Male	8.0 (72.7%)	-	-	-	-
Female	3.0 (27.3%)	-	-	-	-
Fibronectin (pg/ml) (mean±SD)	108.8±6.8	-	-	-	-
Age<40	7.0 (63.6%)	-	-	-	-
Age>40	4.0 (36.4%)	-	-	-	-
Non-Smokers	7.0 (63.6%)	-	-	-	-
Smokers	4.0 (36.4%)	-	-	-	-
Non-Diabetic	11.0 (100.0%)	-	-	-	-

Diabetic	-	-	-	-	-
No of Zones					
0	11.0 (100.0%)	-	-	-	-
**Only Patients with TB**					
**Study Groups**	**0**	**1+**	**2+**	**3+/4+**	***p*-value**
Total (n)	-	26	26	26	
Male	-	20.0 (76.9%)	23.0 (88.5%)	20.0 (76.9%)	*0.476*[1]
Female	-	6.0 (23.1%)	3.0 (11.5%)	6.0 (23.1%)	
Fibronectin (pg/ml) (mean±SD)	-	118.5±26.9	97.7±14.9	91.1±28.7	*< 0.001*[2]
Age<40	-	12.0 (46.2%)	6.0 (23.1%)	8.0 (30.8%)	*0.199*[1]
Age>40	-	14.0 (53.8%)	20.0 (76.9%)	18.0 (69.2%)	
Non-Smokers	-	17.0 (65.4%)	10.0 (38.5%)	18.0 (69.2%)	*0.050*[1]
Smokers	-	9.0 (34.6%)	16.0 (61.5%)	8.0 (30.8%)	
Non-Diabetic	-	19.0 (73.1%)	20.0 (76.9%)	18.0 (69.2%)	*0.822*[1]
Diabetic	-	7.0 (26.9%)	6.0 (23.1%)	8.0 (30.8%)	
No of Zones					0.593[1]
0	-	3.0 (11.5%)	0.0 (0.0%)	2.0 (7.7%)	
1	-	14.0 (53.8%)	9.0 (34.6%)	9.0 (34.6%)	
2	-	6.0 (23.1%)	12.0 (46.2%)	10.0 (38.5%)	
3	-	3.0 (11.5%)	3.0 (11.5%)	3.0 (11.5%)	
4	-	0.0 (0.0%)	1.0 (3.8%)	1.0 (3.8%)	
5	-	0.0 (0.0%)	1.0 (3.8%)	1.0 (3.8%)	

[1]Pearson's Chi-squared test; [2] ANOVA. AFB: Acid-Fast Bacilli.

Table S5: Demographic and clinical characteristics comparison between number of chest X-ray zones in overall study population, controls, and cases

**Overall study population**							
**Study Groups**	**0**	**1**	**2**	**3**	**4**	**5**	***p*-value**
Total	16	32	28	9	2	2	
Male	11.0 (68.8%)	23.0 (71.9%)	26.0 (92.9%)	8.0 (88.9%)	2.0 (100.0%)	1.0 (50.0%)	*0.187*[1]
Female	5.0 (31.2%)	9.0 (28.1%)	2.0 (7.1%)	1.0 (11.1%)	0.0 (0.0%)	1.0 (50.0%)	
Fibronectin (pg/ml) (mean±SD)	107.2±32.2	109.8±29.2	101.5±13.2	93.6±12.0	78.3±6.5	58.3±2.1	*0.026*[2]
Age<40	8.0 (50.0%)	10.0 (31.2%)	9.0 (32.1%)	5.0 (55.6%)	1.0 (50.0%)	0.0 (0.0%)	*0.474*[1]
Age>40	8.0 (50.0%)	22.0 (68.8%)	19.0 (67.9%)	4.0 (44.4%)	1.0 (50.0%)	2.0 (100.0%)	
Non-Diabetic	15.0 (93.8%)	25.0 (78.1%)	17.0 (60.7%)	7.0 (77.8%)	2.0 (100.0%)	2.0 (100.0%)	*0.168*[1]
Diabetic	1.0 (6.2%)	7.0 (21.9%)	11.0 (39.3%)	2.0 (22.2%)	0.0 (0.0%)	0.0 (0.0%)	
AFB 0	11.0 (68.8%)	0.0 (0.0%)	0.0 (0.0%)	0.0 (0.0%)	0.0 (0.0%)	0.0 (0.0%)	*< 0.001*[1]
AFB 1+	3.0 (18.8%)	14.0 (43.8%)	6.0 (21.4%)	3.0 (33.3%)	0.0 (0.0%)	0.0 (0.0%)	
AFB 2+	0.0 (0.0%)	9.0 (28.1%)	12.0 (42.9%)	3.0 (33.3%)	1.0 (50.0%)	1.0 (50.0%)	
AFB 3+/4+	2.0 (12.5%)	9.0 (28.1%)	10.0 (35.7%)	3.0 (33.3%)	1.0 (50.0%)	1.0 (50.0%)	

Non-Smokers	10.0 (62.5%)	19.0 (59.4%)	13.0 (46.4%)	7.0 (77.8%)	1.0 (50.0%)	2.0 (100.0%)	*0.460*[1]
Smokers	6.0 (37.5%)	13.0 (40.6%)	15.0 (53.6%)	2.0 (22.2%)	1.0 (50.0%)	0.0 (0.0%)	
				**Only controls**			
**Study Groups**	**0**	**1**	**2**	**3**	**4**	**5**	***p*-value**
Total	**11**	-	-	-	-	-	-
Male	3.0 (27.3%)	-	-	-	-	-	-
Female	8.0 (72.7%)	-	-	-	-	-	-
Fibronectin (pg/ml) (mean±SD)	108.8±6.8	-	-	-	-	-	-
Age<40	7.0 (63.6%)	-	-	-	-	-	-
Age>40	4.0 (36.4%)	-	-	-	-	-	-
Non-Diabetic	11.0 (100.0%)	-	-	-	-	-	-
Diabetic	-	-	-	-	-	-	-
AFB 0	11.0 (100.0%)	-	-	-	-	-	-
AFB 1+	-	-	-	-	-	-	-
AFB 2+	-	-	-	-	-	-	-
AFB 3+/4+	-	-	-	-	-	-	-
Non-Smokers	7.0 (63.6%)	-	-	-	-	-	-
Smokers	4.0 (36.4%)	-	-	-	-	-	-
				**Only Patients with TB**			
**Study Groups**	**0**	**1**	**2**	**3**	**4**	**5**	***p*-value**
Total	5	32	28	9	2	-2	-
Male	3.0 (60.0%)	23.0 (71.9%)	26.0 (92.9%)	8.0 (88.9%)	2.0 (100.0%)	1.0 (50.0%)	-
Female	2.0 (40.0%)	9.0 (28.1%)	2.0 (7.1%)	1.0 (11.1%)	0.0 (0.0%)	1.0 (50.0%)	-
Fibronectin (pg/ml) (mean±SD)	103.6 (61.2)	109.8 (29.2)	101.5 (13.2)	93.6 (12.0)	78.3 (6.5)	58.3 (2.1)	-
Age<40	1.0 (20.0%)	10.0 (31.2%)	9.0 (32.1%)	5.0 (55.6%)	1.0 (50.0%)	0.0 (0.0%)	-
Age>40	4.0 (80.0%)	22.0 (68.8%)	19.0 (67.9%)	4.0 (44.4%)	1.0 (50.0%)	2.0 (100.0%)	-
Non-Diabetic	4.0 (80.0%)	25.0 (78.1%)	17.0 (60.7%)	7.0 (77.8%)	2.0 (100.0%)	2.0 (100.0%)	-
Diabetic	1.0 (20.0%)	7.0 (21.9%)	11.0 (39.3%)	2.0 (22.2%)	0.0 (0.0%)	0.0 (0.0%)	-
AFB 0	-	-	-	-	-	-	-
AFB 1+	3.0 (60.0%)	14.0 (43.8%)	6.0 (21.4%)	3.0 (33.3%)	0.0 (0.0%)	0.0 (0.0%)	-
AFB 2+	0.0 (0.0%)	9.0 (28.1%)	12.0 (42.9%)	3.0 (33.3%)	1.0 (50.0%)	1.0 (50.0%)	-
AFB 3+/4+	2.0 (40.0%)	9.0 (28.1%)	10.0 (35.7%)	3.0 (33.3%)	1.0 (50.0%)	1.0 (50.0%)	-
Non-Smokers	3.0 (60.0%)	19.0 (59.4%)	13.0 (46.4%)	7.0 (77.8%)	1.0 (50.0%)	2.0 (100.0%)	-
Smokers	2.0 (40.0%)	13.0 (40.6%)	15.0 (53.6%)	2.0 (22.2%)	1.0 (50.0%)	0.0 (0.0%)	-

[1]Pearson's Chi-squared test; [2] ANOVA. AFB: Acid-Fast Bacilli.
